# Supplementary material for: Antimicrobial Consumption in the Livestock Sector in Bhutan: Volumes, Values, Rates, and Trends for the Period 2017–2021
Source: Antibiotics (Basel). 2023 Feb 18;12(2):411. doi: 10.3390/antibiotics12020411 (PMC9952653; doi:10.3390/antibiotics12020411)
Supplement: Supplementary file 1 [file antibiotics-12-00411-s001.zip › antibiotics-2206172-supplementary.pdf]

Table S1. Comparison of the rates of antimicrobial consumption in Bhutan in 2020 with those of 31 European nations

Country	Consumption (tonnes)	Population size (PCU x 1000)	Rate of antimicrobial consumption (mg/PCU)
Austria	43.70	942.30	46.30
Belgium	180.40	1745.30	103.40
Bhutan	0.46	120.88	3.83
Bulgaria	61.10	368.40	166.00
Croatia	22.60	328.90	68.60
Cyprus	48.30	122.60	393.90
Czechia	39.30	699.30	56.30
Denmark	88.70	2384.70	37.20
Estonia	5.70	115.90	49.20
Finland	8.00	494.40	16.20
France	394.40	6964.90	56.60
Germany	684.60	8172.80	83.80
Greece	108.40	1216.50	89.10
Hungary	136.10	801.00	169.90
Iceland	0.50	135.30	3.80
Ireland	102.90	2189.80	47.00
Italy	689.30	3790.40	181.80
Latvia	4.80	157.60	30.80
Lithuania	6.20	302.60	20.50
Luxembourg	1.60	54.40	29.00
Malta	1.70	14.70	116.10
Netherlands	156.40	3114.90	50.20
Norway	4.70	2030.80	2.30
Poland	853.20	4541.70	187.90
Portugal	177.90	1012.00	175.80
Romania	173.70	3003.70	57.80
Slovakia	11.80	228.30	51.90
Slovenia	5.90	176.00	33.30
Spain	1244.50	8067.50	154.30
Sweden	8.70	786.00	11.10
Switzerland	27.70	806.10	34.30
United Kingdom	214.40	7115.20	30.10

Source: European Medicine Agency [8].

Table S2. Comparison by antimicrobial class of the rates of antimicrobial consumption (mg/PCU) in Bhutan in 2020 with those of 31 European nations

Country	Aminoglycosides	Amphenicols	1st and 2nd generation cephalosporins	3rd and 4th generation cephalosporins	Fluoroquinolones	Penicillins	Sulfonamides	Trimethoprim	Tetracyclines	Others*
Austria	1.4	0.4		0.2	0.5	9.9	3.7	0.7	23.5	0.1
Belgium	1.6	1.9	0.3	0.1	0.3	42.5	16.6	3.3	20.5	3.7
Bhutan	0.15	0.01		0.04	0.03	0.2	1.8	0.3	0.5	0.8
Bulgaria	4.3	1.0		0.1	3.7	22.3	6.8	0.8	65.9	0.5
Croatia	2.5	1.5		0.2	2.1	23.1	5.4	1.1	24.8	0.2
Cyprus	5.1	1.2		0.4	2.2	79.4	56.8	11.3	132.0	0.7
Czechia	2.5	0.6	0.1	0.5	1.9	18.3	8.6	1.0	15.5	0.3
Denmark	3.9	0.8		<0.01	<0.01	12.0	3.1	0.6	6.0	1.0
Estonia	3.7	0.4	0.1	0.7	1.1	13.4	4.2	0.8	13.5	0.5
Finland	0.1	0.2	<0.01	<0.01	0.1	8.4	2.8	0.6	3.7	
France	6.7	0.8	0.2	0.0	0.1	8.7	12.3	2.0	18.4	0.5
Germany	2.9	0.8	0.1	0.2	0.8	33.3	7.8	1.1	18.1	1.4
Greece	8.6	1.1		0.2	2.0	15.7	8.1	1.0	43.9	0.6
Hungary	3.3	2.5	0.1	0.5	11.6	51.6	7.0	1.5	57.4	1.2
Iceland	0.6	0.0		<0.01	<0.01	2.8	0.1		0.3	
Ireland	3.2	1.7	0.5	0.2	0.4	12.4	4.6	0.6	19.6	0.6
Italy	7.5	4.9	0.1	0.2	1.2	61.1	26.7	2.6	49.0	1.7
Latvia	4.1	0.2	0.3	0.5	1.5	6.6	1.3	0.3	7.0	0.2
Lithuania	0.5	0.4	0.1	0.1	1.3	6.3	4.1	0.9	2.6	0.3
Luxembourg	3.0	1.0	0.1	0.5	0.8	6.6	2.9	0.6	11.4	0.4
Malta	6.4	2.3	0.1	0.3	4.4	9.5	18.2	3.2	43.9	6.8
Netherlands	0.6	1.5		<0.01	0.1	12.6	8.3	1.5	16.6	
Norway	0.1	0.1		<0.01	<0.01	1.4	0.6	0.1	0.1	<0.01
Poland	7.2	2.2	0.2	0.4	12.9	61.1	8.1	1.6	45.3	2.2
Portugal	5.0	4.4		0.4	7.3	38.9	7.2	1.4	60.4	0.4
Romania	4.7	2.4	<0.01	0.2	5.7	13.7	1.9	0.3	15.4	0.7
Slovakia	3.9	0.3	0.2	0.5	3.4	10.2	6.4	0.9	14.0	1.2
Slovenia	3.6	1.1	0.1	0.2	1.0	15.7	2.4	0.6	7.6	
Spain	10.9	6.4	0.1	0.4	3.7	52.7	12.1	2.1	34.7	1.5
Sweden	0.5			<0.01		6.8	1.9	0.4	0.8	0.2
Switzerland	3.1	0.7	0.1	0.1	0.2	11.0	8.3	0.7	8.4	0.1

United Kingdom	2.5	0.6	0.1		0.1	8.0	2.9	0.6	10.2	0.6

Source: European Medicine Agency [8].
*Metronidazole and nitrofurazone
